# Numerical Simulation of Red Mud Blended Raw Materials in a Precalciner

**DOI:** 10.3390/ma19122500

**Published:** 2026-06-10

**Authors:** Kai Huang, Hongtao Kao

**Affiliations:** College of Materials Science and Engineering, Nanjing Tech University, Nanjing 211816, China; huangkaichang@126.com

**Keywords:** precalciner, red mud, numerical simulation, alternative raw material

## Abstract

The cement industry is a major contributor to global carbon emissions. Therefore, reducing emissions while utilizing industrial wastes is critical for its sustainable development. Red mud, a solid waste byproduct of alumina smelting with main components like SiO_2_, Al_2_O_3_, and CaO, can partially replace limestone as a raw material in cement production. TG-DSC thermal analysis clarified red mud’s three-stage weight loss characteristic during calcination (total weight loss rate of 22.11%), and orthogonal experiments identified calcination temperature as the core factor for its CaO content, with the optimal calcination pretreatment process confirmed (0.075–0.09 mm particle size, 1373 K, 1 h residence time, CaO content up to 21.1%). Based on the results, this study uses ANSYS Fluent 2021 R1 to simulate a TTF-type precalciner, establishing a validated multi-physical field model (all relative errors < 5%) to explore red mud blending ratios of 0%, 2.5%, 5%, 7.5% and 10%. Unlike previous experimental studies, this work uses a CFD model to quantify how red mud blending ratios affect the coupled thermo-chemical environment in a TTF precalciner, revealing a mechanism-driven trade-off among decomposition rate, CO_2_, and NO_x_ that experiments alone cannot capture. Results show red mud slightly alters the internal temperature field and reduces the raw meal decomposition rate. The decomposition rate remains within the industrial acceptable range of 85–95% when the red mud blending ratio is no more than 5%, while further increasing the blending ratio to 7.5% and 10% causes the decomposition rate to drop below 85%. Therefore, a blending ratio of 5% is recommended, which balances waste utilization, decomposition rate, and emission reduction, providing solid technical support for red mud’s large-scale use in cement production.

## 1. Introduction

The cement industry is an important component of the basic materials sector in economic construction. However, this industry is associated with substantial energy consumption and carbon dioxide (CO_2_) emissions [1,2]. Cement manufacturing contributes approximately 50% of industrial CO_2_ emissions within the global cement sector and 7% of worldwide anthropogenic carbon emissions [3]. Cement production also generates large amounts of pollutants such as nitrogen oxides (NO_x_), sulfur dioxide (SO_2_), and particulate matter (PM) [4]. These emissions severely impact air quality and ecological environment. A pressing challenge for the cement industry is reducing energy consumption and pollutant emissions while maintaining production capacity. There are several ways to reduce emissions in the cement industry today, such as alternative raw materials, alternative fuels, and CO_2_ capture [5]. Use of alternative raw materials is considered one of the most promising approaches for energy conservation and emission reduction.

In traditional cement production, limestone raw material decomposes while releasing large amounts of CO_2_ at high temperatures during the calcination process [6]. Industrial bypass products or wastes can be partial substitutes for traditional limestone raw materials. This reduces carbon emissions from the thermal decomposition of carbonate minerals and consumption of natural mineral resources.

Red mud is a solid waste from the industrial alumina smelting process. It is the residue after extraction of alumina from bauxite by strong alkalis, such as sodium hydroxide (NaOH). The vast red mud resources represent huge development potential. As an alternative material, red mud holds significant advantages in the cement industry. Its chemical composition primarily includes oxides like SiO_2_, CaO, Fe_2_O_3_, Al_2_O_3_, Na_2_O, TiO_2_, and K_2_O. The content of calcium compound (e.g., CaO, CaCO_3_) in red mud is relatively low compared to high-calcium industrial wastes. This limits its potential for large-scale raw material replacement. However, dual objectives can be achieved if red mud in small portions is used to replace some traditional raw materials. It helps alleviate environmental pressures while enabling resource utilization of waste and production cost optimization.

Currently, numerous scholars have conducted research on red mud as alternative raw materials.

Zheng Hexin et al. found that clinker compressive strength can be increased by 3–4 MPa with red mud in the cement raw meal system [7]. The underlying mechanism involves the moderately elevated alkali content in red mud.

TSAKIRIDIS et al. demonstrated that Portland cement clinker conforming to ASTM standards can be produced with red mud addition ≤ 3.5% [8]. Its mineral phase composition (e.g., C_3_S, C_2_S) is similar to conventional clinker, and its 28-day compressive strength reaches 52–55 MPa, which is comparable to ordinary clinker. This provides experimental evidence for the large-scale resource utilization of red mud in the cement industry.

Wang Xiao et al. prepared cement clinker by calcination at 1450 °C with raw materials of dealkalized red mud from Bayer process, sandstone, limestone, and fly ash [9,10]. They measured the specific activity of radionuclide across different process stages (calcination, hydration) using XRD, a gamma spectrometer and other techniques.

Dong Lumin et al. replaced a portion of cement clinker through thermal treatment of red mud-fly ash composite system and prepared low-carbon cementitious materials [11]. They found that nepheline in red mud decomposed into active Al_2_O_3_ and SiO_2_ after calcination at 600 °C, while the risk of efflorescence was reduced with effective alkali decreased from 6.8% to 1.2%.

Xia Ruijie et al. prepared HBCSA clinker through synergistic activation from primary raw materials of red mud (rich in Al_2_O_3_, Fe_2_O_3_) and desulfurized gypsum (CaSO_4_·2H_2_O) [12]. They creatively utilized red mud and desulfurized gypsum to prepare special cement, promoting a “dual-waste linkage” technology.

Liu et al. utilized the numerical simulation method to simulate the heat transfer, mass transfer, and chemical reaction processes of coal gangue in the precalciner, and analyzed the gas–solid two-phase flow field under different operating conditions.

Liu Y et al. set up test conditions with different municipal sludge addition ratios [13]. The results showed that municipal sludge combustion had good stability, and replacing part of the fuel did not affect the operation of the precalciner.

Liu R.P et al. carried out three-dimensional numerical simulation of an industrial-scale cement kiln precalciner to explore the effects of different ammonia blending ratios on fuel burnout characteristics and NO formation when ammonia is used as a carbon-free alternative fuel [14].

Qu, Q.Q.Q et al. designed a precalciner suitable for high substitution ratio multi-component heterogeneous alternative fuels [15]. Actual operation showed that the heat substitution rate at the kiln tail of the precalciner reached 80%.

Wang X.Q et al. studied the technology of combining coal gangue as an alternative fuel with oxygen-enriched combustion in a TTF-type precalciner [16].

Wang J et al. constructed a mathematical and physical model using ANSYS FLUENT 2021 R1 software to simulate the combustion process of sludge with different moisture contents and dosages in a TTF-type precalciner [17].

Prateek S et al. proposed using ash-free producer gas generated by gasification of refuse-derived fuel as an alternative fuel [18].

Venkata V.K et al. adopted a microstructure-oriented modeling method to study the influence of lignin and lignin-biomass mixed fuels on the heat transfer of limestone calcination [19].

Wang B et al. carried out CFD simulation on a TTF-type precalciner to explore the differences in coal combustion under O_2_/N_2_ and O_2_/CO_2_ atmospheres [20].

Grzegorz B et al. adopted the Euler–Lagrange two-phase flow method to carry out simulation on the co-combustion of solid recovered fuel (SRF) in an AT-type precalciner [21].

Amila K et al. analyzed the calcination process in the precalciner when RDF replaced 50% of coal through CFD simulation [22].

Roger H et al. focused on the problem of natural gas replacing heavy fuel oil (HFO) for precalciner combustion; when the factory attempted to directly replace the fuel, problems such as excessive temperature at the preheater outlet and excessive CO emissions occurred [23].

The above review demonstrates that most existing studies on the application of red mud as an alternative raw material in cement production are mainly limited to experimental investigations. Nevertheless, the cement precalciner is a large-scale reactor involving intricate physical and chemical processes, including fuel combustion and coupled raw material decomposition, such as limestone decomposition and interfacial reactions between red mud and other raw materials. Experimental approaches alone are insufficient to guide practical cement production. In particular, red mud derived from different bauxite sources exhibits unstable chemical compositions, and its high alkali content may easily induce operational risks such as preheater scaling. At present, research concerning the partial replacement of conventional raw materials with red mud inside precalciners remains scarce, which greatly restricts the large-scale resource utilization of red mud in the cement industry.

To fill the above research gaps, this study extends CFD-based alternative material assessment from fuels to raw materials by systematically simulating red mud blending in a TTF-type precalciner—an application domain that remains absent in previous numerical studies. Unlike experimental works that report optimal blending ratios without clarifying underlying mechanisms, this work quantitatively identifies a clear trade-off between environmental benefit (CO_2_/NO_x_ reduction) and production efficiency (CaCO_3_ decomposition rate), determining the critical threshold (5%) beyond which the industrial decomposition requirement (85%) is violated. Furthermore, this study reveals that red mud suppresses NO_x_ not simply by reducing coal consumption, but by altering local oxygen distribution and avoiding ultra-high-temperature zones—a mechanism not previously reported for red mud co-processing in precalciners.

Based on the above, this study first analyzes the calcination performance of red mud to identify the key influencing factors. On this basis, a three-factor and four-level orthogonal experiment is designed to systematically explore the effects of particle size, calcination temperature, and residence time on the calcination behavior, and to determine the maximum calcium oxide content after red mud calcination. Furthermore, computational fluid dynamics (CFD) numerical simulation is adopted to investigate the calcination process of red mud-blended raw meals in a TTF-type precalciner. The influences of different red mud blending ratios on the temperature field, species distribution, raw material decomposition rate, and NO_x_ emission characteristics within the precalciner are comprehensively analyzed. The primary objective is to determine the optimal red mud blending ratio under feasible operating conditions. The findings are expected to provide a theoretical basis and technical support for the practical engineering application of red mud in cement precalciners, and further promote the dual goals of solid waste resource recycling and carbon emission reduction in the cement industry.

## 2. Simulation Methods and Mathematical Models

### 2.1. Fundamental Conservation Equation

Flowing fluids are always governed by fundamental physical conservation laws. These laws include the conservation of mass, energy, and momentum [24]. Governing equations are their mathematical representations.

(1) Law of mass conservation states that the rate of change of mass within the control volume equals the net rate of mass flow into the control volume. Its mathematical expression is:

On one hand, it effectively mitigates issues such as unstable clinker quality and surging fuel consumption caused by temperature anomalies. On the other hand, by proactively predicting temperature trends, it significantly enhances production efficiency, reduces overall energy consumption, and facilitates the intelligent and green transformation of the cement industry [25].

(1) $$\frac{\partial \rho }{\partial t}+\frac{\partial \left(\rho v_{i}\right)}{\partial x}+\frac{\partial \left({\rho v}_{j}\right)}{\partial y}+\frac{\partial \left(\rho v_{k}\right)}{\partial z}=0$$

For steady-state flow, the density remains constant over time, hence:

(2) $$\frac{\partial \left(\rho v_{i}\right)}{\partial x}+\frac{\partial \left({\rho v}_{j}\right)}{\partial y}+\frac{\partial \left(\rho v_{k}\right)}{\partial z}=0$$

where ρ represents the fluid density (kg/m^3^); *t* is time (s); and $v_{i}$, $v_{j}$, and $v_{k}$ are the velocity vector components in the x, y, and z directions, respectively (m/s).

(2) Law of momentum conservation states that the rate of momentum change for a fluid element equals the total external force exerted on it. The component expressions along three coordinate axes are given below:

(3) $$\frac{\partial (\rho u)}{\partial t}+div\left(\rho u\vec{v}\right)=S_{u}+div\left(\mu gradu\right)-\frac{\partial p}{\partial x}$$

(4) $$\frac{\partial (\rho v)}{\partial t}+div\left(\rho v\vec{v}\right)=S_{v}+div\left(\mu gradv\right)-\frac{\partial p}{\partial y}$$

(5) $$\frac{\partial (\rho w)}{\partial t}+div(\rho w\vec{v})=S_{w}+div\left(\mu gradw\right)-\frac{\partial p}{\partial z}$$

where $\vec{v}$ represents the fluid velocity vector; $S_{u}$, $S_{v}$, and $S_{w}$ represents the generalized source terms in the x, y, and z directions; ρ is the fluid density; p is the static pressure; and μ is the coefficient of viscosity.

(3) Law of energy conservation states that the rate of energy increase within a control volume equals the net heat flux into the volume plus the work done on the volume by body and surface forces. Its mathematical expression is:

(6) $$\frac{\partial (\rho h)}{\partial t}+\frac{\partial \left(\rho u_{i}h\right)}{\partial x_{i}}=div\left(\lambda gradT\right)-pdiv\vec{u}+\phi +S_{h}$$

where λ denotes fluid thermal conductivity; $S_{h}$ is the internal heat source; ϕ is the viscous dissipation function; and T represents temperature (K).

### 2.2. Gas-Phase Turbulence Model and Particle Motion Model

Most fluid flows are laminar or turbulent. It is predominantly turbulent flow within a precalciner. There are also swirl and secondary flows given the complexity of fluid flows in the precalciner. This study employs a swirl-modified realizable *k*-*ε* model [26], which effectively simulates complex flows like secondary and swirling flows, where *k* is the turbulent kinetic energy, and *ε* is the turbulent dissipation rate. The swirl-modified realizable *k*-*ε*; model equations are as follows:

(7) $$\frac{\partial \left(\rho k\right)}{\partial t}\;+\;\frac{\partial \left(\rho k\mu_{i}\right)}{\partial x_{i}}\;=\;\frac{\partial }{\partial x_{j}}\left[\left(\mu +\frac{\mu_{t}}{\sigma_{k}}\right)\frac{\partial k}{\partial x_{j}}\right]\;+\;G_{k}\;+\;G_{b}\;-\;\rho \varepsilon \;-\;Y_{M}\;+\;S_{k}$$

(8) $$\frac{\partial \left(\rho \varepsilon \right)}{\partial t}+\frac{\partial \left(\rho \varepsilon \mu_{i}\right)}{\partial \mathrm{xi}}=\frac{\partial }{\partial x_{j}}\left[\left(\mu \;+\frac{\mu_{t}}{\sigma_{\varepsilon }}\right)\frac{\partial \varepsilon }{\partial x_{j}}\right]-C_{2\varepsilon }\rho \frac{\varepsilon^{2}}{k}+C_{1\varepsilon }\frac{\varepsilon }{k}\left(G_{k}+C_{3\varepsilon }G_{b}\right)+S_{\varepsilon }$$

where $G_{k}$ is the turbulent kinetic energy generated by the mean velocity gradients; $G_{b}$ is the turbulent kinetic energy due to buoyancy; $Y_{M}$ is the effect of fluctuating dilatation in compressible turbulence; $C_{1\varepsilon }$, $C_{2\varepsilon }$, and $C_{3\varepsilon }$ are empirical constants; $\sigma_{k}$ and $\sigma_{\varepsilon }$ are the turbulent Prandtl numbers for turbulent kinetic energy and dissipation rate; and $S_{k}$ and $S_{\varepsilon }$ are defined source terms.

The discrete phase model (DPM) was used to simulate particle motion within the furnace. The simulation was based on the Lagrangian specification of a stochastic trajectory model [27]. The particle motion equation in the stochastic trajectory model is as follows:

(9) $$\frac{du_{p}}{\mathrm{dt}}\;={\;F}_{D}\left(u-u_{p}\right)\;+\;\frac{g_{x}\left(\rho_{p}\;-\;\rho \right)}{\rho_{p}}\;+\;F_{x}$$

where $F_{D}\left(u\;-\;u_{p}\right)$ is the drag force per unit particle mass (N/kg); *u* is the gas phase velocity (m/s); $\rho_{p}$ is the particle density (kg/m^3^); $g_{x}$ is the gravitational acceleration (m/s^2^); and $F_{x}$ is the sum of other forces acting on the particle besides gravity (N).

### 2.3. Particle Combustion Model

The kinetics/diffusion-limited combustion model was employed to simulate combustion of residual char after volatile matter release [28]. The char combustion rate is affected by both the diffusion rate (*D*_0_) of oxygen molecules to the char surface and the reaction rate (*K*) between the char and oxygen molecules upon arrival at the surface [29]. The expressions are as follows:

(10) $$\frac{dm_{p}}{\mathrm{dt}}\;=\;-A_{p}P_{\mathrm{OX}}\frac{D_{0}K}{D_{0}+K}$$

(11) $$D_{0}={\;C}_{1}\frac{{\left[\left(T_{P}+T_{\infty }\right)/2\right]}^{0.75}}{d_{p}}$$

(12) $$K=C_{2}e^{-(E_{2}/RT_{p})}$$

where $A_{p}$ is the particle surface area; $P_{\mathrm{OX}}$ is the partial pressure of oxygen in the surrounding gas; $d_{p}$ is the particle diameter; and $T_{P}$ and $T_{\infty }$ are the initial particle temperature and the temperature of surrounding gas, respectively.

### 2.4. Raw Material Decomposition

Only the decomposition of CaCO_3_ was considered in simulating raw meal decomposition. The species transport and finite-rate/eddy-dissipation models were used to simulate CaCO_3_ decomposition. The species transport conservation equation is as follows:

(13) $$\frac{\partial }{\partial_{t}}\left(\rho Y_{i}\right)\;+\;\nabla \left(\rho \vec{v}Y_{i}\right)\;=\;-\nabla j_{i}\;+\;R_{i}\;+\;S_{i}$$

where $Y_{i}$ is the mass fraction of species i; $R_{i}$ is the net production rate by chemical reaction; $S_{i}$ is the additional rate from dispersed phase and user-defined sources; and $J_{i}$ is the diffusion flux of species. The reaction equation of CaCO_3_ decomposition is as follows:

(14) $$\mathrm{CaC}O_{3}\left(s\right)\;⟶\;\mathrm{CaO}\left(s\right)+CO_{2}\left(g\right)\;∆H\;=\;+178\mathrm{KJ}/\mathrm{mol}$$

The decomposition rate of raw meal is calculated as follows:

(15) $$\omega \;=\;\frac{{\mathrm{CaCO}}_{3\mathrm{inlet}}-\mathrm{CaC}O_{3\mathrm{outlet}}}{\mathrm{CaC}O_{3\mathrm{inlet}}}\;\times \;100\%$$

where ${\mathrm{CaCO}}_{3\mathrm{inlet}}$ is the total mass flow rate of CaCO_3_ at the inlet; $\mathrm{CaC}O_{3\mathrm{outlet}}$ is the mass flow rate of CaCO_3_ at the outlet.

### 2.5. NO_x_ Generation Model

There are three models for NO_x_ compound formation in Fluent: prompt NO_x_, thermal NO_x_, and fuel NO_x_. There are very small amounts of prompt and thermal NO_x_ generated in the precalciner. Accordingly, thermal and fuel NO_x_ models were used to account for NO_x_ formation in the precalciner.

### 2.6. Solution Methods and Assumptions

Fluent software is based on the finite volume method (FVM), also known as control volume method (CVM). Hence, the governing equations were solved by FVM. It is a numerical discretization technique based on integral conservation laws. The SIMPLE algorithm was selected, and Second Order Upwind scheme was used.

(1) The simulations are performed under steady-state conditions because industrial precalciners operate under continuous and stable conditions without significant time-dependent variations in the main operating parameters.
(2) Radiation heat transfer is solved using the P-1 radiation model. The P-1 model is particularly suitable for optically thick, large-scale domains. It offers acceptable accuracy for engineering simulations while significantly reducing computational cost and improving numerical efficiency. Considering the practical operating characteristics and computational demands of the TTF-type precalciner, the P-1 model is adopted in this work.
(3) Coal devolatilization is modeled using the two-competing-rate (Kobayashi) model, which accounts for the release of volatile matter at different heating rates. Char combustion is described by the kinetics/diffusion-limited model presented in Section 2.3 (10–12), assuming that the char surface reaction is controlled by both chemical kinetics and oxygen diffusion. The volatile matter is assumed to consist of CO, H_2_, CH_4_, and other hydrocarbons, and its combustion is modeled using the eddy-dissipation model (EDM) with a single-step global reaction.
(4) The particle size distributions of raw meal, pulverized coal, and red mud are described by the Rosin–Rammler function. The characteristic particle diameter and spread parameter for each material are set according to experimental test data. Specifically:

Pulverized coal: minimum 5 μm, maximum 90 μm, and mean 65 μm.

Raw materials (including red mud-blended mixtures): minimum 20 μm, maximum 60 μm, and mean 45 μm.

(5) Red mud is incorporated into the raw meal on a mass-substitution basis at blending ratios of 0%, 2.5%, 5%, 7.5%, and 10%. The composition of the blended raw meal is the mass-weighted average of the original raw meal and red mud. Red mud’s physical properties (density, specific heat, decomposition behavior) are derived from TG-DSC analysis (Section 2.7), while the decomposition kinetics of the blended raw meal follow the same first-order model as pure CaCO_3_, with parameters from orthogonal experiments (Section 2.8). Catalytic effects of red mud on CaCO_3_ decomposition are not considered.
(6) Additional assumptions: All particles (coal, raw meal, red mud) are assumed spherical and non-deformable. The lumped capacitance method is used, assuming uniform temperature within each particle due to high solid-phase thermal conductivity. The gas phase is assumed to be an ideal gas mixture with temperature-dependent specific heat and thermal conductivity. The precalciner walls are assumed adiabatic (no heat loss), reflecting the typical thermal insulation in industrial precalciners. These assumptions are consistent with previous CFD studies on cement precalciners and provide a reasonable balance between model fidelity and computational efficiency.

### 2.7. Thermal Analysis Test

X-ray fluorescence (XRF) was used to determine the chemical compositions of raw meal and red mud. The test was performed using a Panalytical Zetium XRF spectrometer (Malvern Panalytical, Almelo, The Netherlands).

Thermogravimetry-differential scanning calorimetry (TG-DSC) was employed to analyze the thermal behavior of red mud. The test was carried out on a TA SDT Q600 instrument (TA Instruments, New Castle, DE, USA) over a temperature range from 30 °C to 1400 °C with a heating rate of 20 °C/min.

**Figure 1:**
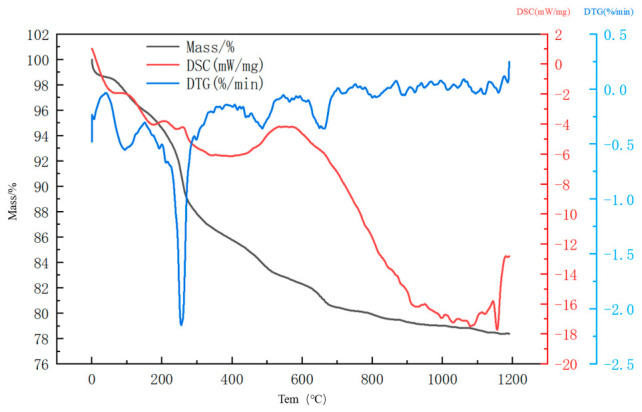
TG-DSC Thermal Analysis Curve.

A comprehensive thermal analysis of the red mud was performed with thermogravimetric analysis (TGA) and differential scanning calorimetry (DSC) as shown in Figure 1.

The red mud maintained a weight loss state during heating, which could be divided into three stages: 30–377.6 °C, 377.6–902.3 °C, and 902.3–1200 °C. In the range of 30–377.6 °C, the TG curve began to decrease noticeably with significant weight loss, mainly attributed to the gradual release of volatile substances such as physically adsorbed water on the sample surface and a small amount of organic impurities. The weight loss rate in the first stage was relatively high, with a weight loss percentage of 14.32%. In the range of 377.6–902.3 °C, the TG curve decreased, indicating that the loss of chemically bound water mainly resulted from the decomposition of Al(OH)_3_: 2Al(OH)_3_ → Al_2_O_3_ + 3H_2_O and the release of CO_2_ from the decomposition of CaCO_3_: CaCO_3_ → CaO + CO_2_. The evaporation of structural water and the release of CO_2_ led to a substantial weight loss of 6.76% in the second stage. Within 902.3–1200 °C, the weight loss was caused by the reaction between Al_2_O_3_ and CaO formed after dehydration of red mud to produce C_3_A or C_2_A. No obvious rapid mass change was observed, and the weight loss was the smallest at 1.03%. The total weight loss during the entire heating process was 22.11%.

From the DSC curve, it was found that organic matter and impurities underwent slow and continuous oxidative exothermic processes during heating.

### 2.8. Orthogonal Test

Considering the factors that affect the calcination behavior of red mud in the calciner, three independent variables with no mutual interference were adopted, each having four levels. The factors and levels are as follows: red mud particle size (˂0.075 mm, 0.075–0.09 mm, 0.09–0.18 mm, ˃0.18 mm), calciner temperature (773 K, 973 K, 1173 K, 1373 K), and material residence time (0 h, 0.5 h, 1 h, 1.5 h).

Since no standard orthogonal array is available for three factors and four levels, a more compatible design was adopted using the L16(4^5^) orthogonal array to establish the three-factor and four-level orthogonal experimental scheme. The specific experimental factors, levels and scheme are shown in Table 1 and Table 2.

### 2.9. Test Results and Analysis

**Figure 2:**
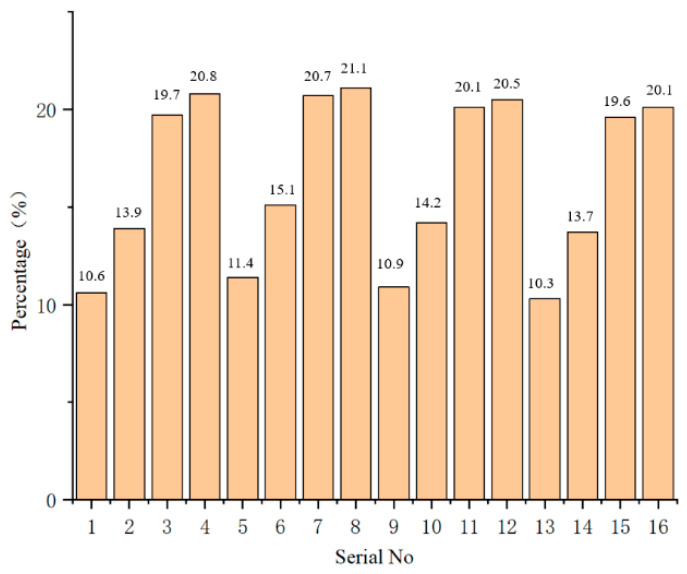
Orthogonal Experimental Results.

The Orthogonal test results are shown in Figure 2.

Through the comparison of the percentage of CaO content obtained from the orthogonal experiments, it can be concluded that when the raw material particle size is 0.075–0.09 mm, the calcination temperature is 1373 K, the residence time reaches 1 h, and the CaO content in the sample is the highest, reaching 21.1%.

### 2.10. Range Analysis of Orthogonal Experiment

Range calculation was performed on the results corresponding to different levels of each factor, and the range results are presented in Table 3. In the range analysis of the orthogonal experiment, a larger range value indicates that the factor has a more significant influence on the experimental results. Thus, the order of significance of each factor’s influence on the results can be determined.

For the factor-level analysis, the optimal level of each factor can be determined by the Ki values (sum of experimental results at different levels of a factor) and ki values (average results at different levels of a factor): 0.075–0.09 mm, 1373 K, and 1.5 h.

It should be clarified that the theoretical optimal combination obtained by range analysis is A_2_B_4_C_4_.

However, the 1 h residence time mentioned in the text represents the optimal condition among the 16 actual orthogonal experimental groups, which achieved the highest measured CaO content in the experiments. This discrepancy arises because range analysis predicts an extrapolated optimum beyond the tested level combinations, whereas the measured optimum is limited to the actual experimental runs. Two considerations support the selection of 1 h as the practical optimum. First, the ANOVA results indicate that residence time has no statistically significant effect on CaO content. Second, from an industrial perspective, a shorter residence time reduces energy consumption, making it economically preferable.

### 2.11. Analysis of Variance

The orthogonal test analysis of variance is shown in Table 4. It can be concluded that temperature is the main factor affecting the percentage of CaO.

For the orthogonal analysis, the calciner environment was simulated in a muffle furnace by heating from room temperature to the target temperature at a rate of 10 K/min. The two analytical methods yield basically consistent conclusions. Specifically, temperature is always the most important factor, with the highest range and F-value, so it should be strictly optimized and controlled. Particle size and residence time show the lowest significance in both methods and have no obvious influence on the experimental results.

The orthogonal experiment determines the optimal calcination pretreatment for red mud (0.075–0.09 mm, 1373 K, 1 h), achieving a maximum CaO content of 21.1%. These conditions are applied to pretreat red mud before CFD simulations. Furthermore, the experimental results provide CFD input parameters and compositional data for blended raw meals. Thus, the orthogonal experiment directly informs the CFD simulations with experimentally validated red mud properties.

### 2.12. Discussion on Simplifications

The current model focuses on CaCO_3_ decomposition as the dominant reaction in the precalciner, while other potential reactions involving red mud components are not considered. This simplification is justified for the following reasons: (1) The main oxides in red mud (SiO_2_, Al_2_O_3_, Fe_2_O_3_) remain largely inert at precalciner temperatures (~1200 K); their significant reactions with CaO (e.g., formation of C_2_S, C_3_A, C_4_AF) typically occur above 1400 K in the rotary kiln. (2) The red mud blending ratio is limited to ≤10%, and its CaO content is only 17.86%, so the additional mass of reactive oxides is minor relative to the raw meal. (3) The primary function of the precalciner is CaCO_3_ decomposition; clinker mineral formation is the focus of the downstream rotary kiln.

Nevertheless, the following limitations should be acknowledged: (1) Alkali components (Na_2_O, K_2_O) in red mud may volatilize at high temperatures and condense on cooler surfaces, potentially contributing to preheater scaling—a long-term operational risk not captured by steady-state simulations. (2) Low-melting-point phases may form in the Fe_2_O_3_–Na_2_O system, which could influence coating behavior. (3) Potential catalytic or inhibitory effects of transition metal oxides on CaCO_3_ decomposition are not included. These aspects are considered beyond the scope of the present study but warrant future investigation, particularly for industrial-scale long-term operation.

## 3. Basic Information

### 3.1. Geometrical Model and Grid

**Figure 3:**
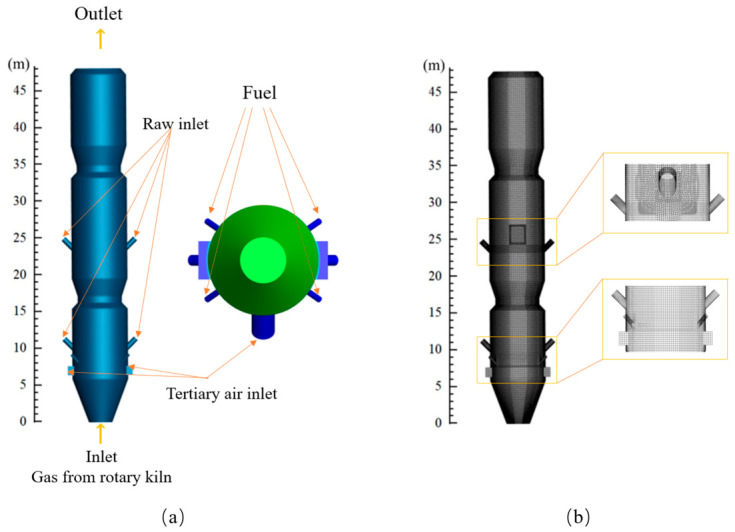
Schematic diagram of the precalciner: (**a**) geometric model and (**b**) mesh.

The study subject is a TTF-type precalciner from a cement plant. Figure 3 illustrates its geometric model, composed of three cylindrical sections, two constricted neck sections, and a lower conical section. The precalciner is 45.8 m high, with varying diameters across its height. Specifically, the constricted neck and lower conical sections have smaller diameters, while the upper, middle, and lower cylindrical sections have larger diameters. The maximum diameter is 7.2 m. This structural design facilitates the spouting effect for gas flow in the furnace. The lower conical section is 5.6 m high, and the lower, middle and upper cylindrical sections have a height of 9.1 m, 13 m, and 9.2 m, respectively. Two raw meal inlet pipes are symmetrically distributed on both the middle and lower cylindrical sections. This staged feeding arrangement increases the decomposition rate of raw meal while contributing to the uniform temperature distribution in the furnace. At a furnace height of 3.5 m, two tertiary air ducts are symmetrically arranged, above which are four symmetrically arranged coal injection pipes, forming a 40-degree angle with the ducts.

A structured hexahedral grid structure was utilized for precalciner simulation. A grid independence study was conducted to separate simulation results from the grid number. This process validates the accuracy of simulation results and helps determine the appropriate grid number. Five grid numbers were selected (823,324, 933,996, 1,064,400, 1,095,458 and 1,166,580). Table 5 compares the predicted average outlet temperatures and the measured outlet temperatures. The values are the closest when the grid number is 1,064,400. Changes in the predicted temperature with further increase in grid number are negligible. Hence, a grid configuration with 1,064,400 cells was selected for subsequent simulations in Figure 3.

### 3.2. Chemical Composition and Boundary Conditions

Table 6 presents the chemical composition of raw meal and red mud.

Correct boundary conditions in CFD software are crucial for accurate numerical simulation results. They are predefined initial parameters, and their proper configuration directly determines the deviation between predicted and measured values. Table 7 shows the boundary conditions in the simulation.

Pulverized coal particles are assumed spherical with a density of 1400 kg/m^3^, specific heat capacity of 1100 J/(kg·K), volatile matter release temperature of 400 °C, particle swelling coefficient of 1.1, and initial temperature of 337 K. The calcium carbonate particles have a density of 2800 kg/m^3^, specific heat capacity of 856 J/(kg·K), thermal conductivity of 2.25 W/(m·K), and initial temperature of 1040 K. The main components of flue gas from the rotary kiln are 11.3 vol% CO_2_, 1.0 vol% O_2_, 0.5 vol% CO and 87.2 vol% N_2_.

### 3.3. Model Verification

**Figure 4:**
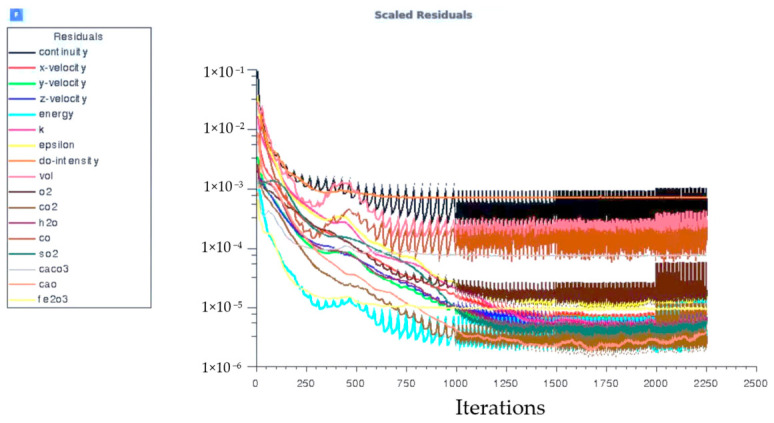
Residual curve of the calculation.

To verify the reliability of simulation results, predicted outlet parameters were compared against field measurement data from the thermal calibration of the precalciner, as listed in Table 8. The deviations between simulated and experimental data are below 5%. After 1500 steps, the residuals of energy, k and ε equations all drop below 10^−4^, and the residual curves level off without obvious upward or downward drift after reaching a low order of magnitude. Figure 4 shows the residual curves from the model calculations. Hence, the simulation results are reliable.

All boundary condition data listed in Table 7 (including inlet velocities, temperatures, pressures, mass flow rates, and turbulence intensities) were obtained from on-site thermal calibration measurements at the collaborating cement plant under normal operating conditions. The measurement procedures for outlet temperature and flue gas composition are described in detail below, and the verification results in Table 8 confirm that the simulated outlet parameters agree well with the measured data, with all relative errors below 5%. This falls within the engineering acceptable range for precalciner simulations, demonstrating that the boundary conditions are physically reasonable and that the model is reliable for subsequent parametric studies.

## 4. Simulation Results and Discussion

### 4.1. Combustion Simulation Under Different Red Mud Blending Ratios

#### 4.1.1. Influence on the Temperature Field

**Figure 5:**
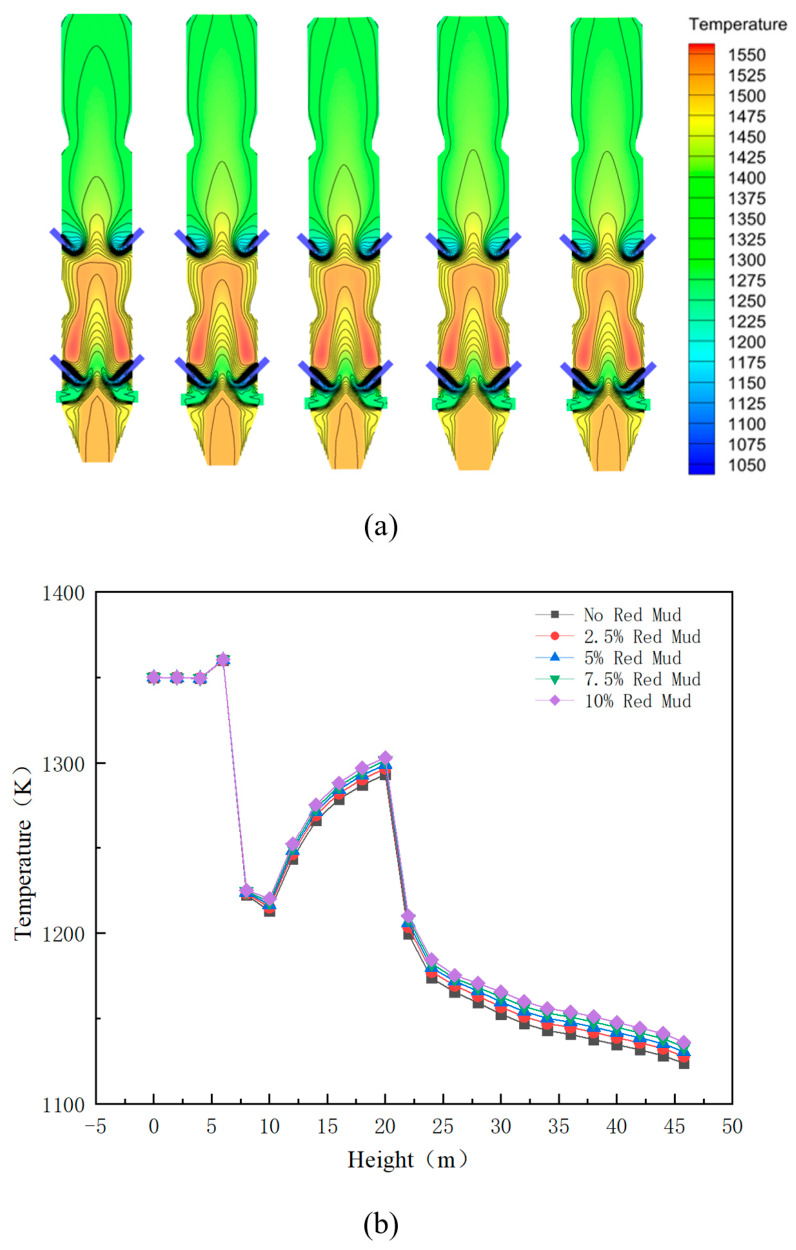
(**a**) Temperature plots on Z = 0 section and (**b**) average gas temperature at different red mud blending ratios.

Figure 5 shows the temperature contour plots on the Z = 0 section and the average gas temperature at different heights in the precalciner when blending different proportions of red mud. The temperature field in the furnace does not change significantly with the proportion of red mud. High-temperature zones are consistently concentrated in the main combustion zone between the upper and lower raw meal inlet pipes and in the conical section. The temperature is relatively low in the middle and upper cylindrical sections. Notably, as the blending ratio of red mud increases, the high-temperature zone enlarges significantly and the maximum temperature rises significantly in the main combustion zone. The local maximum temperature in the furnace rises from 1487 K without red mud blending to 1500 K, 1502 K, 1506 K, and 1510 K at blending ratios of 2.5%, 5%, 7.5%, and 10%, respectively, representing an increase of 13 K, 15 K, 19 K, and 27 K. This indicates that the substitution of partial raw materials with red mud leads to a change in the maximum temperature inside the furnace. Additionally, as the blending ratio of red mud increases, the temperature in the middle and upper cylindrical sections of the precalciner gradually rises, and the outlet temperature of the precalciner also increases progressively with the increase in the blending ratio.

#### 4.1.2. Influence on Raw Material Decomposition

**Figure 6:**
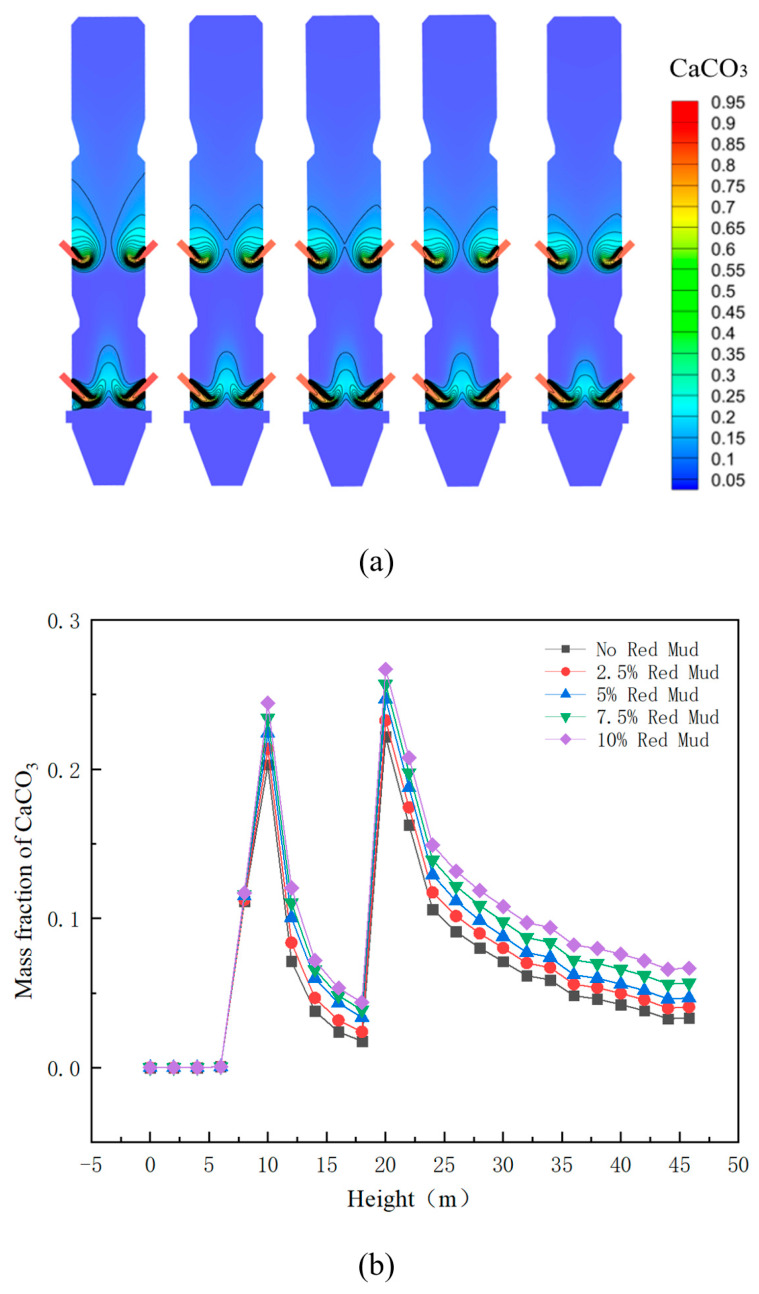
(**a**) CaCO_3_ distribution on Z = 0 section and (**b**) average CaCO_3_ mass fraction along the precalciner height at different red mud blending ratios.

**Figure 7:**
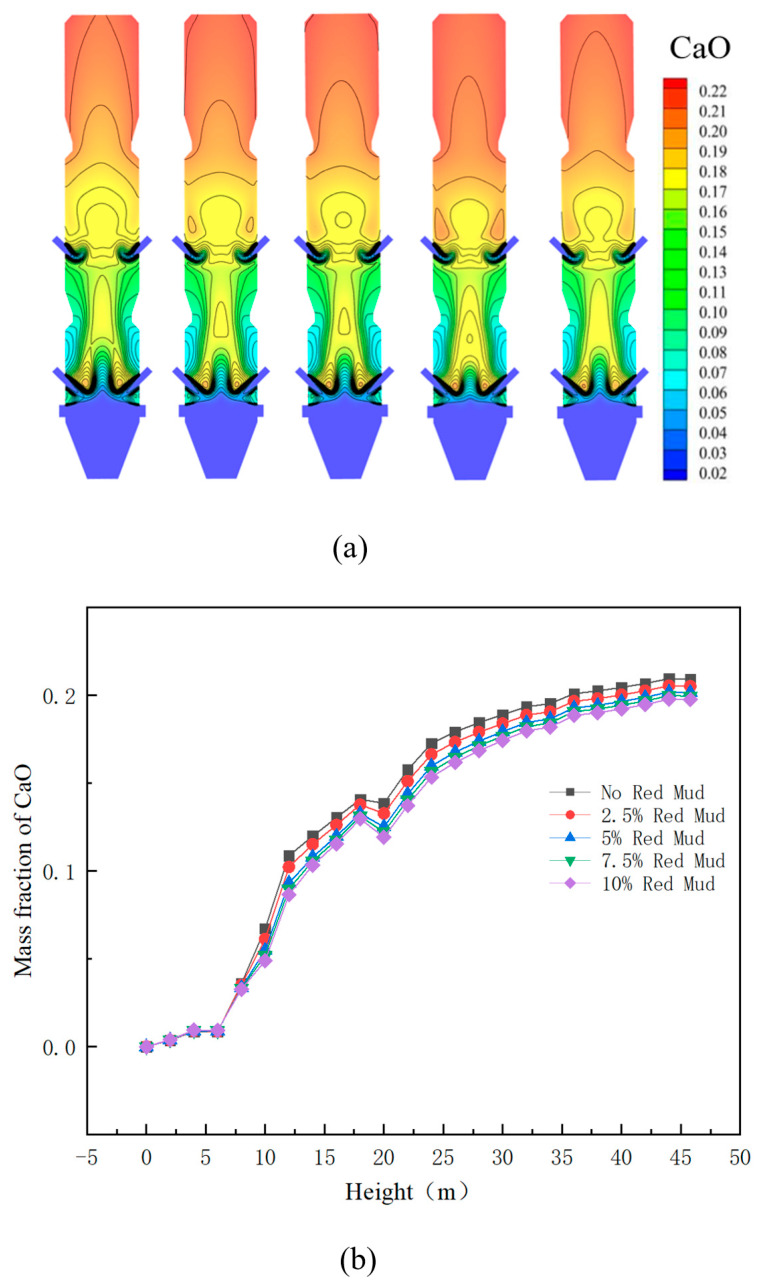
(**a**) CaO distribution on Z = 0 section and (**b**) average CaO mass fraction along the precalciner height at different red mud blending ratios.

Figure 6 and Figure 7 illustrate the concentration distribution on the Z = 0 section and the average mass fractions of CaCO_3_ and CaO at different heights in the precalciner when blending different proportions of red mud. High CaCO_3_ concentrations are primarily located near the upper and lower raw meal inlet pipes, while high CaO concentrations are distributed in the upper cylindrical section.

On the Z = 0 section, CaCO_3_ and CaO concentrations exhibit opposite trends. Raw meal entering from the lower pipe is carried into the main combustion zone after intense heat exchange with high-temperature tertiary air and flue gas. A large amount of heat released by pulverized coal combustion is absorbed to decompose most CaCO_3_. In this process, CaCO_3_ concentration decreases rapidly, while CaO concentration increases rapidly. The upper portion of the raw meal is decomposed mainly by heat from the ascending hot flue gas and the continued combustion of unburned char. The heat intensity is relatively low, as is the decomposition rate. In contrast, raw meal entering from the upper pipe decomposes as it rises, but at a lower decomposition rate compared to the main combustion zone. This process sees decreasing CaCO_3_ concentration and increasing CaO concentration. The decomposition is also slow due to relatively low heat intensity, but complete decomposition is still achieved due to longer residence time in the main combustion zone compared to the lower portion. Overall, the lower portion decomposes rapidly due to direct exposure to the high-temperature main combustion zone, while the upper portion decomposes due to indirect heat from the hot flue gas. This arrangement effectively utilizes the heat at different locations in the furnace, while contributing to the optimal thermal efficiency and efficient synergy between material reactions.

Notably, when different proportions of red mud are blended, the variation trend of the average mass fraction of CaCO_3_ in the furnace with height is consistent with that during pulverized coal combustion alone. The overall average mass fraction of CaCO_3_ gradually increases, resulting in a decrease in the raw meal decomposition rate. According to calculations, the decomposition rates of CaCO_3_ in the raw meal at the outlet are 91.22%, 89.53%, 86.02%, 83.10%, and 80.81%. Considering that the decomposition rate of raw meal at the precalciner outlet is generally required to be controlled within 85–95% in actual production, it is recommended that the blending ratio of raw materials with red mud should be 5%, which can not only ensure a relatively high substitution rate but also meet the requirement of raw meal decomposition rate.

#### 4.1.3. Influence on Composition Fields

**Figure 8:**
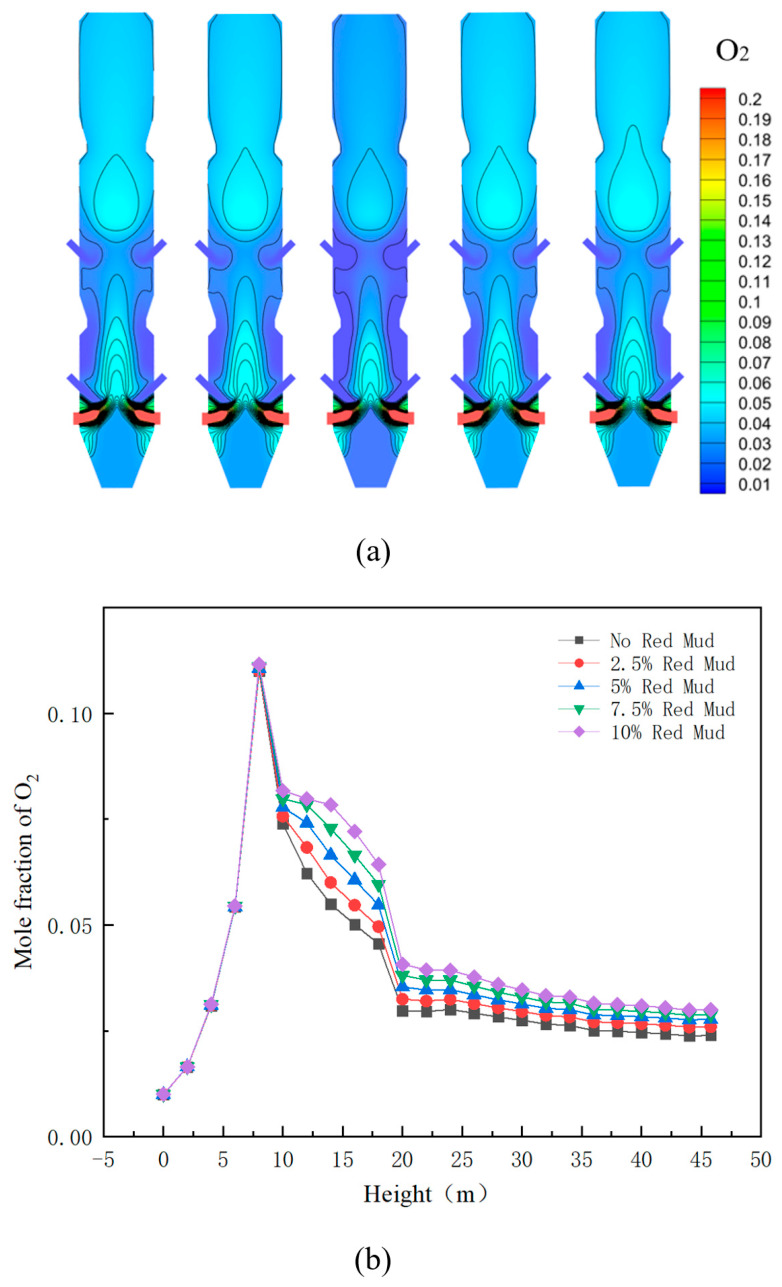
(**a**) O_2_ distribution on Z = 0 section and (**b**) average O_2_ mole fraction along the precalciner height at different red mud blending ratios.

**Figure 9:**
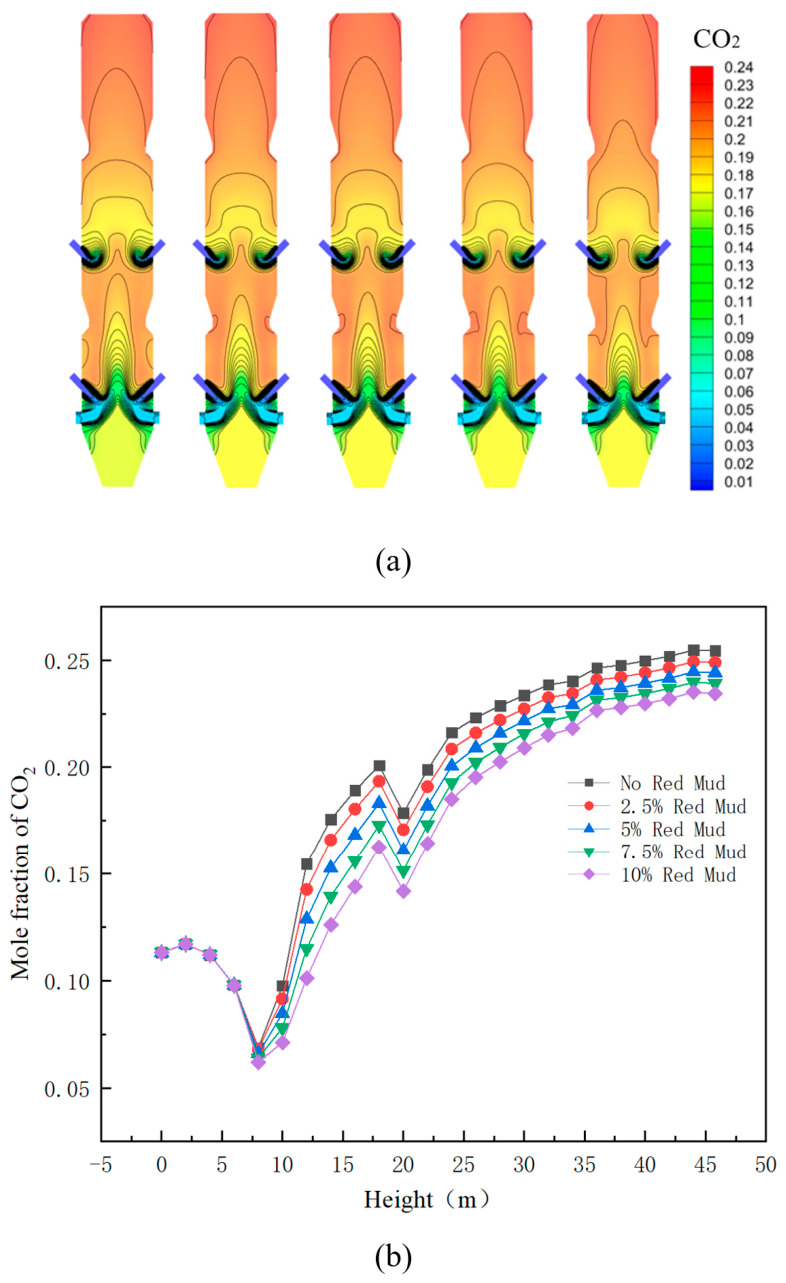
(**a**) CO_2_ distribution on Z = 0 section and (**b**) average CO_2_ mole fraction along the precalciner height at different red mud blending ratios.

Figure 8 and Figure 9 show the concentration distribution on the Z = 0 section in the furnace under pulverized coal combustion conditions and the average mole fractions of O_2_ and CO_2_ at different heights in the precalciner when blending different proportions of red mud. As shown in Figure 8, high O_2_ concentrations are located near the tertiary air inlets, where the air serves as the primary combustion source. O_2_ concentration decreases with increasing height due to rapid consumption in the main combustion zone. CO_2_ is released from both coal combustion and raw meal decomposition, resulting in high CO_2_ concentrations in the main combustion zone. In the middle and upper cylindrical sections, O_2_ continues to decline slowly due to residual char combustion, while CO_2_ further accumulates from ongoing decomposition and burnout of unburned char.

The reduction in CO_2_ emissions with increasing red mud blending ratio is attributed to two primary factors. First, the replacement of limestone by red mud directly reduces the amount of CaCO_3_ entering the precalciner. Based on mass balance, the CaCO_3_ input decreases proportionally with the blending ratio, leading to a theoretical CO_2_ reduction from carbonate decomposition of approximately 1.8% per 2.5% red mud addition. Second, the reduced heat demand (due to less endothermic CaCO_3_ decomposition) lowers coal consumption. The coal feed rate was adjusted to maintain the same outlet temperature, and the resulting reduction in coal-derived CO_2_ contributes an additional ~0.5% per 2.5% red mud addition. Dilution effects (changes in flue gas volume) and flow-field modifications have minor impacts (<0.2%) on the overall CO_2_ concentration change and are not the main drivers.

#### 4.1.4. Influence on NO_x_ Compounds

**Figure 10:**
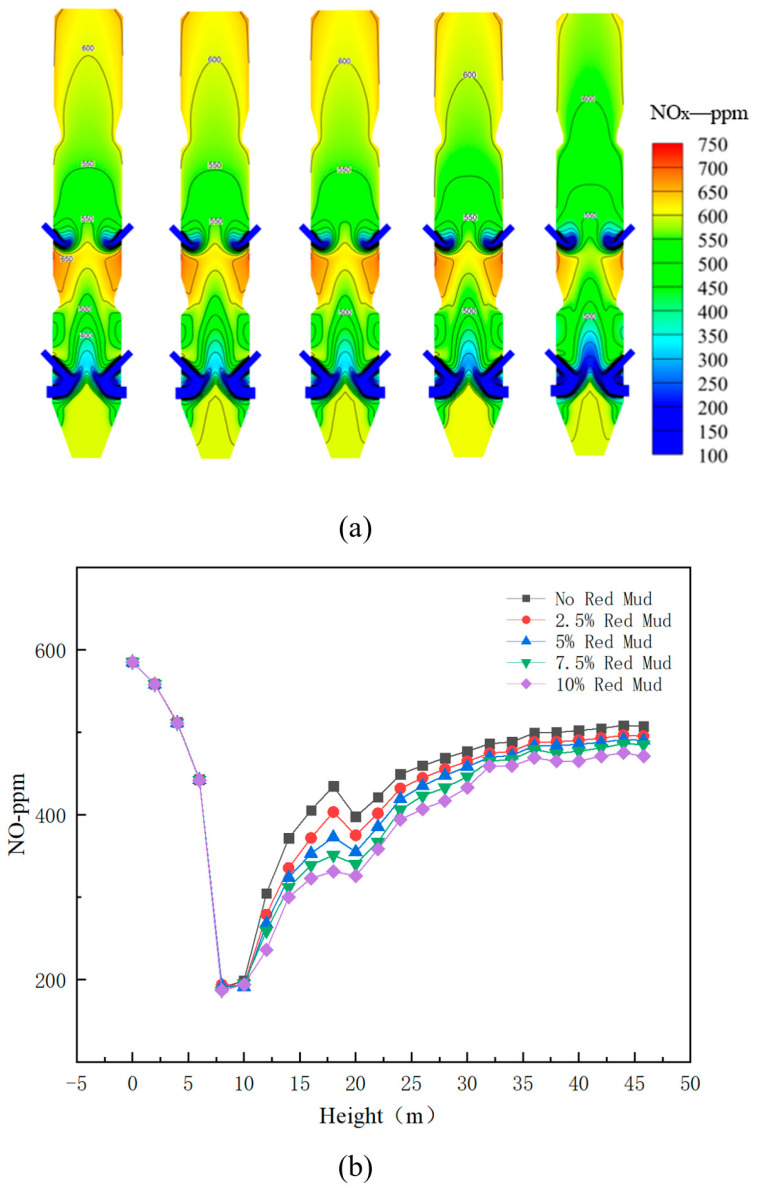
(**a**) NO distribution on Z = 0 section and (**b**) NO concentration (ppm) along the precalciner height at different red mud blending ratios.

Figure 10 shows the NO distribution on the Z = 0 section and the average NO concentration at different heights under various red mud blending ratios. NO_x_ in the precalciner primarily includes fuel NO_x_ from coal combustion and thermal NO_x_ from kiln flue gas (measured at approximately 583 ppm). Local high NO concentrations are located in the main combustion zone between the upper and lower raw meal inlet pipes, where sufficient O_2_ promotes intense coal combustion, generating substantial fuel NO_x_ and a small amount of thermal NO_x_. The NO distribution in the conical section, which contains NO from the rotary kiln, does not change significantly across blending ratios. However, as the blending ratio increases, the high-NO region in the main combustion zone shrinks, and the local peak NO concentration decreases.

The total heat demand declines with increasing red mud proportion, reducing the need for excessive coal combustion and avoiding local ultra-high-temperature zones, which further suppresses thermal NO_x_. Notably, the NO reduction is not a dilution effect: the absolute NO mass flow rate at the outlet decreases from 0.152 g/s to 0.141 g/s, confirming a real reduction. In terms of outlet concentrations, as the blending ratio increases from 0% to 10%, the average NO concentration decreases from 507 ppm to 471 ppm (a reduction of 36 ppm at 10%). However, at blending ratios above 5%, the further NO reduction is offset by an unacceptable drop in decomposition rate (<85%). Potential catalytic NO reduction by metal oxides in red mud is not considered in the current model and warrants further investigation.

The observed NO reduction is primarily driven by two mechanisms. First, the local maximum temperature in the main combustion zone increases only modestly from 1487 K to 1502 —well below the 1800 K threshold where thermal NO_x_ becomes dominant. Thus, the contribution of reduced thermal NO_x_ is minor. Second, as shown in Figure 8, the O_2_ distribution shifts with red mud addition: oxygen becomes less concentrated in the pulverized coal combustion zone, reducing the probability of fuel-bound nitrogen being oxidized to NO_x_. To quantitatively assess the contributions of thermal and fuel NO_x_ mechanisms to the observed NO reduction, three separate simulations were performed under identical operating conditions for the red mud blending case:

Case 1 (Full model): Both thermal and fuel NO_x_ formation models were activated, yielding the total outlet NO concentration.

Case 2 (Fuel NO_x_ only): The thermal NO_x_ formation was deactivated, allowing the simulation to calculate the NO concentration originating solely from fuel-bound nitrogen.

Case 3 (Thermal NO_x_ only): The fuel NO_x_ formation was deactivated, allowing the simulation to calculate the NO concentration generated solely by the thermal mechanism.

Due to the reversible chemical interactions and mutual consumption between thermal and fuel NO_x_ species, the total NO reduction cannot be attributed to either mechanism by simple arithmetic subtraction. Therefore, the reduction magnitude was estimated between Case 2 and Case 1. Using this method, the contribution of fuel NO_x_ suppression to the overall NO reduction was estimated to be approximately 70–80%.

### 4.2. Discussion

The recommendation of 5% red mud blending is primarily based on maintaining the raw meal decomposition rate above the industrial threshold of 85%. However, practical optimization for industrial application requires consideration of additional factors:

At 5% blending, the outlet NO concentration decreases from 507 ppm to 495 ppm (a 2.4% reduction), and CO_2_ emissions are reduced due to lower limestone input. Higher blending ratios (7.5–10%) offer greater emission reductions (e.g., 10% blending reduces NO to 471 ppm, a 7.1% reduction), but at the cost of unacceptable decomposition rate (<85%).

The temperature field remains stable across all blending ratios, with no dramatic local hot spots or cold zones introduced by red mud addition. The maximum temperature increases moderately from 1487 K (0%) to 1502 K (5%), which is within the material tolerance limits.

As discussed in Section 2.12, alkali components (Na_2_O, K_2_O) in red mud may volatilize and condense on cooler surfaces, potentially contributing to preheater scaling. This risk increases with higher blending ratios. At 5%, the additional alkali input is limited (red mud contains only trace amounts of Na_2_O and K_2_O), but long-scale validation is still warranted.

The impact of red mud on clinker mineralogy (e.g., C_3_S, C_2_S formation) and cement properties (e.g., setting time, compressive strength) was not evaluated in this study, as these processes occur downstream in the rotary kiln. Previous experimental studies suggest that blending ratios up to 5% do not significantly degrade clinker quality. However, composition-specific validation is recommended for each cement plant.

In summary, 5% represents the optimal blending ratio under the constraint of maintaining decomposition rate ≥ 85%. For plants with different raw meal chemistry, kiln configurations, or product specifications, the optimal ratio may vary, and pilot-scale testing is advised.

## 5. Conclusions

This work shifts research from red mud experimental characterization toward quantitative multi-field CFD simulation, advancing insights into red mud as an alternative raw meal in precalciners. Its core novelty lies in quantifying the inherent trade-off between emission reduction gains and raw meal decomposition performance—a mechanism difficult to obtain through experiments alone. Key findings are summarized as follows:

TG-DSC analysis shows three-stage mass loss (total 22.11%) from 30 °C to 1200 °C. Orthogonal experiments identify calcination temperature as the dominant factor affecting CaO content. The optimal conditions (0.075–0.09 mm, 1373 K, 1 h) yield a CaO content of 21.1%.

Blending red mud does not alter the overall temperature distribution. The local maximum temperature rises modestly from 1487 K (0%) to 1502 K (5%) and 1510 K (10%), within material tolerance limits.

A clear downward trend in the raw meal decomposition rate is observed with the increase in red mud blending ratio: 91.22% (0%), 89.53% (2.5%), 86.02% (5%), 83.10% (7.5%), and 80.81% (10%). Taking into account the conventional industrial criterion that the raw meal decomposition rate at the precalciner outlet should be maintained within the range of 85–95%, a 5% red mud blending ratio is determined as the optimal proportion.

CO_2_ reduction comes from reduced carbonate decomposition (~1.8% per 2.5% red mud addition) and lower coal consumption (~0.5% per 2.5% addition). NO reduction is primarily driven by fuel NO_x_ suppression (70–80% of total reduction) due to shifted O_2_ distribution, rather than thermal NO_x_ reduction. The outlet NO concentration decreases from 507 ppm (0%) to 471 ppm (10%), with a real reduction confirmed by mass flow rate decrease (0.152 g/s → 0.141 g/s at 5%).

From the simulation results, a clear contradictory trade-off exists with the increase in red mud addition. Introducing red mud can effectively cut down fossil coal consumption and lower CO_2_ emissions, realizing resource utilization of industrial solid waste. Nevertheless, excessive red mud (7.5% and 10%) absorbs calcination heat, directly suppressing CaCO_3_ decomposition and dropping rate below the industrial qualified threshold of 85%. There is an optimal blending window within 5% red mud dosage, where the decomposition rate meets production requirements while achieving considerable emission reduction. Exceeding this proportion means the environmental gain cannot offset the production loss, making the balance between emission reduction benefit and calcination performance the core constraint for industrial application of red mud co-processing.

This study reveals the intrinsic mechanism by which red mud blending affects the multi-physical fields and reaction processes in the precalciner. Through quantitative simulation, the optimal red mud blending ratio for the TTF-type precalciner is determined to be 5%. This ratio not only realizes efficient resource utilization of red mud solid waste but also maintains a stable raw meal decomposition rate, achieving synergistic reduction of CO_2_ and NO_x_ emissions. The results provide clear technical guidance for the large-scale engineering application of red mud in the cement industry and offer a general numerical simulation approach for the green and low-carbon transformation of cement production. The above results are obtained based on numerical simulations. Further pilot-scale and industrial tests are still required to verify the practical applicability and operational stability of red mud co-processing in actual cement precalciner production.

## Figures and Tables

**Table 1:** Factor level table.

Actor	Particle Size A/mm	Temperature B/K	Residence Time C/h
Level	<0.075	773	0
	0.075–0.09	973	0.5
	0.09–0.18	1173	1
	>0.18	1373	1.5

**Table 2:** Orthogonal experimental table.

Serial No	Size (mm)	Temperature (K)	Residence Time (h)
1	<0.075	773	0
2	<0.075	973	0.5
3	<0.075	1173	1
4	<0.075	1373	1.5
5	0.075–0.09	773	0.5
6	0.075–0.09	973	0
7	0.075–0.09	1173	1.5
8	0.075–0.09	1373	1
9	0.09–0.18	773	1
10	0.09–0.18	973	1.5
11	0.09–0.18	1173	0
12	0.09–0.18	1373	0.5
13	>0.18	773	1.5
14	>0.18	973	1
15	>0.18	1173	0.5
16	>0.18	1373	0

**Table 3:** Results of range analysis.

Factor		A	B	C
Percentage of CaO	K_1_	55.5	10.6	55.1
	K_2_	57	44.9	55.6
	K_3_	56.6	82.1	55.6
	K_4_	55	86.5	57.8
	k_1_	13.875	2.65	13.775
	k_2_	14.25	11.225	13.9
	k_3_	14.15	20.525	13.9
	k_4_	13.75	21.625	14.45
	Range (R)	0.5	18.975	0.55
Priority	BAC			
Combination	A_2_B_4_C_4_			

**Table 4:** ANOVA results and significance analysis.

Factor	SS	f	Ms	F	Critical F	Significance
A	0.6519	3	0.2173	3.372	F_0.05_(3,6) = 4.76	Not significant
B	948.9569	3	316.319	77.217	F_0.01_(3,6) = 9.78	Significant
C	1.0919	3	0.364	0.6869	F_0.05_(3,6) = 4.76	Not significant
S*_e_*	951.7094	6	158.6182	/	/	/
Sum	1902.41	15	/	/	/	/

where SS is the sum of squares, f is the degrees of freedom for the factor, Ms is the mean square, F is the test statistic, and Se is the standard error.

**Table 5:** Predicted vs. measured average outlet temperature with different grid numbers.

Grid Number (Cell)	823,324	933,996	1,064,400	1,095,458	1,166,580
Predicted Temp. (K)	1125	1142	1160	1158	1157
Measured Temp. (K)	1184	1184	1184	1184	1184
Error	4.98%	3.54%	2.03%	2.19%	2.28%

**Table 6:** Chemical composition of raw meal and red mud.

Sample	SiO_2_	Al_2_O_3_	Fe_2_O_3_	CaO	MgO	LOI
Raw Meal (%)	11.76	3.69	2.76	42.83	1.30	35.28
Red Mud (%)	18.1	23.41	28.66	17.86	0.10	5.25

**Table 7:** Boundary conditions in simulation.

Parameter	Flue Gas Inlet	Upper Tertiary Air Inlet	Lower Tertiary Air Inlet	Raw Meal Inlet	Pulverized Coal Inlet	Flue Gas Outlet
Velocity (m/s)	40	35	35		25	
Hydraulic Diameter (m)	3	1.5	1.5	0.7	0.4	3
MFR (kg/s)				10		
Temp. (K)	1350	1300	1300	1040	330	1150
Turbulence Intensity	5	7	7	7	5	5
Pressure (pa)	−500	−720	−500	−720	−500	1250

**Table 8:** Simulated vs. measured outlet parameters.

Parameter	Simulated	Measured	Error (%)
Outlet Temp. (K)	1160	1184	2.03
CO_2_ (%)	25.44	26.2	2.99
O_2_ (%)	2.39	2.44	2.09
Raw Meal Decomposition Rate (%)	91.22	90.12	1.21
NO (ppm)	507	521	2.76

## Data Availability

The original contributions presented in this study are included in the article. Further inquiries can be directed to the corresponding author.

## Abbreviations

The following abbreviations are used in this manuscript:

TG-DSC	Thermogravimetry-Differential Scanning Calorimetry
TTF	Triple-Tangential-Flow Furnace
C_2_S	2CaO·SiO_2_
C_3_A	3CaO·Al_2_O_3_
C_4_AF	4CaO·Al_2_O_3_·Fe_2_O_3_
